# Navigating the poliovirus threat: a call for training and resources in Gaza

**DOI:** 10.3389/fpubh.2024.1487525

**Published:** 2025-01-08

**Authors:** Marwa Saleh, Rajaa Musleh, Shafiq Yousef, Collins Ouru, Fatma Al Atawna

**Affiliations:** MedGlobal, Chicago, IL, United States

**Keywords:** public health, polio best practice, infectious disease, humanitarian access, healthcare worker (HCW)

## Discussion of recent findings on poliovirus in Gaza

The detection of vaccine-derived poliovirus type 2 in sewage samples from Gaza in July 2024, followed by the one confirmed case of acute flaccid paralysis in a 10-month-old in August highlighted the urgency of addressing polio transmission risks (1, 2). Despite the conflict, approximately 94% of the 591,714 targeted children under ten were vaccinated with a second dose of nOPV2 by October 2024, marking a significant achievement. However, northern Gaza remained a concern, with an 88% coverage rate leaving < 10,000 children unvaccinated in hard-to-reach areas like Jabalia, Beit Lahiya, and Beit Hanoun (3). Furthermore, ongoing conflict and insecurity in Gaza continue to severely disrupt health, water, and sewage systems, creating conditions conducive to additional disease transmission. MedGlobal clinics are also documenting increasing numbers of hepatitis A and pediatric skin infections including scabies.

## Implications of healthcare worker preparedness

A rapid self-assessment conducted by MedGlobal between 18^th^ August to 21^st^ August 2024 aimed to evaluate healthcare workers' preparedness for a potential polio outbreak in Gaza. Utilizing a snowball sampling method, the survey reached a diverse group of frontline healthcare workers, including nurses, doctors, midwives, nutritionists, and psychologists. The survey focused on three key areas: knowledge of detection, diagnosis and management of polio, specific training needs, and resource accessibility.

The results, based on 83 responses, revealed that 55% (46) of healthcare workers were unfamiliar with managing polio in children, while only 17% (14) felt confident in their knowledge. This indicates a critical need for enhanced training in early detection, diagnosis, and management of polio symptoms, as well as infection prevention and control measures.

The survey further highlighted that 69% (57) of respondents felt under-equipped to respond effectively to a polio outbreak. The predominant reason cited for this lack of preparedness was insufficient resources and tools. Additionally, 71% (58) of respondents expressed a need for training in early detection and diagnosis of polio symptoms, while 66% (54) requested training on infection prevention and control measures.

These findings suggest that Gaza's healthcare system is poorly equipped to detect and manage polio highlighting an urgent need for integrated preparedness and response efforts. There is an urgent need for targeted capacity building activities and resource mobilization to enhance the healthcare workers preparedness in Gaza. While vaccinating will ensure that prevention efforts are maximized, however, the collapsed health care conditions, require additional consideration of worse case scenario where healthcare workers must be more confident in detection and management of potential polio in children and adults. These findings also support the need to do similar assessments for other infectious diseases with outbreak potential, such as cholera and measles.

## Recommendations

1. Training and Capacity Building: Implement comprehensive training programs focused on the early detection, diagnosis, and management of polio for all frontline workers especially in northern Gaza. This must take into consideration the limited internet connectivity, the fluctuating security situation across Gaza with no permanent safe houses for staff and the pre-existing burden on health care staff amidst the worsening health status of the local population.

2. Resource Mobilization: Secure and distribute necessary medical supplies and equipment, including protective equipment and diagnostic tools, to healthcare facilities.

3. Ongoing Assessment and Support: Conduct regular assessments of healthcare worker preparedness and resource availability to swiftly identify and address emerging gaps.

4. Empower community health workers to report health events and increase awareness of outbreak symptoms.

The importance of these measures cannot be overstated, as timely interventions will support further prevention of polio resurgence, and provide lessons learned for other infectious disease detection and management from a healthcare worker preparedness perspective in Gaza.

## Data Availability

The original contributions presented in the study are included in the article/Supplementary material, further inquiries can be directed to the corresponding author.
